# Comparison of Erosion Behavior and Particle Contamination in Mass-Production CF_4_/O_2_ Plasma Chambers Using Y_2_O_3_ and YF_3_ Protective Coatings

**DOI:** 10.3390/nano7070183

**Published:** 2017-07-14

**Authors:** Tzu-Ken Lin, Wei-Kai Wang, Shih-Yung Huang, Chi-Tsung Tasi, Dong-Sing Wuu

**Affiliations:** 1 Department of Materials Science and Engineering, National Chung Hsing University, Taichung 40227, Taiwan; alexlin19781121@hotmail.com (T.-K.L.); d100066018@mail.nchu.edu.tw (C.-T.T.); 2 Department of Materials Science and Engineering, Da-Yeh University, Changhua 51591, Taiwan; wk@mail.dyu.edu.tw; 3 Department of Industrial Engineering and Management, Da-Yeh University, Changhua 51591, Taiwan; syh@mail.dyu.edu.tw; 4 Center for Advanced Industry Technology and Precision Processing, National Chung Hsing University, Taichung 40227, Taiwan

**Keywords:** yttrium fluoride (YF_3_), yttrium oxide (Y_2_O_3_), atmospheric plasma spraying (APS), particle contamination

## Abstract

Yttrium fluoride (YF_3_) and yttrium oxide (Y_2_O_3_) protective coatings prepared using an atmospheric plasma spraying technique were used to investigate the relationship between surface erosion behaviors and their nanoparticle generation under high-density plasma (10^12^–10^13^ cm^−3^) etching. As examined by transmission electron microscopy, the Y_2_O_3_ and YF_3_ coatings become oxyfluorinated after exposure to the plasma, wherein the yttrium oxyfluoride film formation was observed on the surface with a thickness of 5.2 and 6.8 nm, respectively. The difference in the oxyfluorination of Y_2_O_3_ and YF_3_ coatings could be attributed to Y–F and Y–O bonding energies. X-ray photoelectron spectroscopy analyses revealed that a strongly fluorinated bonding (Y–F bond) was obtained on the etched surface of the YF_3_ coating. Scanning electron microscopy and energy dispersive X-ray diffraction analysis revealed that the nanoparticles on the 12-inch wafer are composed of etchant gases and Y_2_O_3_. These results indicate that the YF_3_ coating is a more erosion-resistant material, resulting in fewer contamination particles compared with the Y_2_O_3_ coating.

## 1. Introduction

Silicon-based ceramics have been extensively used in semiconductor plasma processing equipment as plasma-facing materials, due to their hardness, high wear resistance, dielectric strength, high corrosion resistance, and chemical stability [1,2]. They are used mainly as a shield to protect the ceramic parts inside etchers or chemical vapor deposition reactor chambers from corrosion caused by fluorocarbon corrosive gases such as CF_4_, CHF_3_, C_4_F_6_, and C_2_F_6_ [3,4,5]. These materials interact with plasma and are eroded, resulting in the production of contaminant particles on the wafer. As integrated circuits continue to scale down with wider use of high-density plasma for wafer processing, the particles generated in the plasma processing equipment cause serious problems, such as short current in integration circuit and lower production yield [6,7]. In order to solve this problem, yttrium oxide (Y_2_O_3_) was adopted as plasma-facing inner wall materials in plasma processing equipment because their plasma erosion resistance values are much higher than those of conventional SiO_2_ coatings [8,9,10]. Mass-production factories have found that the Y_2_O_3_ inner walls have problems with significant erosion and particle generation [11]. Under fluorine-based plasma processing, a thin top carbonaceous polymer reaction layer has been identified depending on the etching conduction and the etched materials [12,13,14]. The polymer layer thickness is determined using polymer deposition and its removal rate, and consumption rate in substrate etching. This polymer layer etching rate is affected by the plasma incident ion kinetic energy. Some volatile etching products are produced during the etching process, such as carbon oxide, carbon oxyfluorides, and silicon fluorides [15]. Unlike silicon-based materials, the yttrium-based material etching mechanism is not fully understood. Yttrium fluoride (YF_3_) coatings ave recently attracted substantial attention because of their high plasma erosion resistance, preventing the generation of fluoride particles on the chamber wall surface, reducing particulate contamination [16]. Thus, the YF_3_ coating might be a new plasma-facing material that produces fewer contamination particles. The Y_2_O_3_ and YF_3_ coatings were deposited using atmospheric plasma spraying (APS). In this study, the mechanism of formation of yttrium oxyfluoride film and their particle trajectories in industrial plasma processing tools have been examined. Moreover, we compared the etching behavior of Y_2_O_3_ and YF_3_ coatings and their compounds under fluorocarbon plasma. The surface morphology, chemical reactions on the etched surface, microstructure, and particle contaminations of Y_2_O_3_ and YF_3_ coatings were investigated.

## 2. Materials and Methods

**Figure 1:**
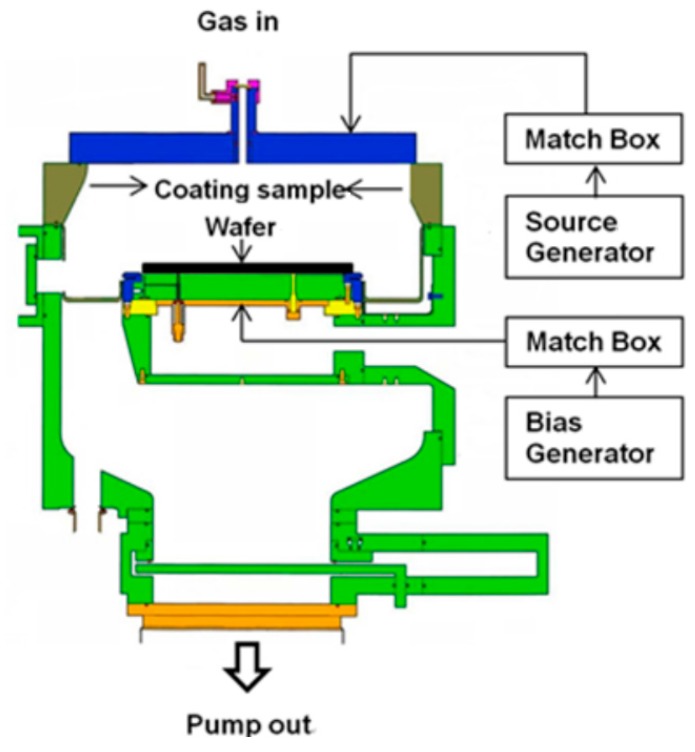
Schematic illustration of the cross section of inductively coupled plasma (ICP) etching tool.

Commercially available YF_3_ powders (25–50 μm, 99.99%, Shin-Etsu Chemical, Tokyo, Japan) and Y_2_O_3_ powders (25–50 μm, 99.99%, Shin-Etsu Chemical, Tokyo, Japan) were used as the spraying materials. YF_3_ and Y_2_O_3_ coatings were prepared using APS with an F4-MB plasma gun (Sulzer Metco, Orelikon, Pfaeffikon, Switzerland). An alloy aluminum (A6061) substrate was used for the experiment. The specimen had a size of 400 mm^2^ and a thickness of 20 mm. Before APS, the substrate was treated with sandblasting. The sandblasting material was SiO_2_. Acetone was used to clean the substrate. The stand-off distance was adjusted to 10 cm. The Ar and H_2_ gas cylinders were opened when the air compressor was initiated. The Ar flow rate, H_2_ flow rate, system voltage, gun movement rate, and feed rate were set to 45 L/min, 6 L/min, 50 V, 10 cm/s, and 15 g/min, respectively. The YF_3_ and Y_2_O_3_ spraying parameters are shown in Table 1. The erosion behaviors of both protective coatings were performed using an inductively coupled plasma (ICP) etcher (LAM 2300 Metal) under the routine plasma etching process; i.e., the same bias power and processing gases (CF_4_ and O_2_). High-density CF_4_/O_2_ plasma with electron densities on the order of 10^12^ to 10^13^ cm^−3^ has been produced. Figure 1 shows a schematic diagram of the ICP etcher system employed in this study. The etch process details are shown in Table 2. Three-hundred millimeter blanket wafers with chemical vapor deposition titanium nitride layer/Si-substrate were prepared to evaluate the integrated circuit defective performance after dry etching process measured by Surfscan SP3 (Surfscan SP3, KLA-Tencor Corporation, Milpitas, CA, USA). 

The surface morphology, microstructure, and elemental analysis of these coating samples were conducted using scanning electron microscopy (SEM, S-3000H, Hitachi, Tokyo, Japan) coupled with energy dispersive X-ray diffraction (EDX) and transmission electron microcopy (TEM, H-600, Hitachi, Tokyo, Japan). The composition of these samples were examined by X-ray photoelectron spectroscopy (XPS, PHI 5000 VersaProbe, ULVAC-PHI, Kanagawa, Japan) using a monochromatic Al Kα X-ray source at a passing energy of 20 eV with a spot size of 650 μm, then the sample surface was etched using focused argon ions sputtering to investigate the chemical compositional depth profile (ThermoScientific K-Alpha). A fitting software program (Thermo Fisher Scientific, Inc., Waltham, MA, USA) was used to deconvolute the photoelectron spectrum resulting from the core energy levels of Yttrium 3d states to estimate the contributions from bonding with fluorine elements. 

## 3. Results and Discussion

**Figure 2:**
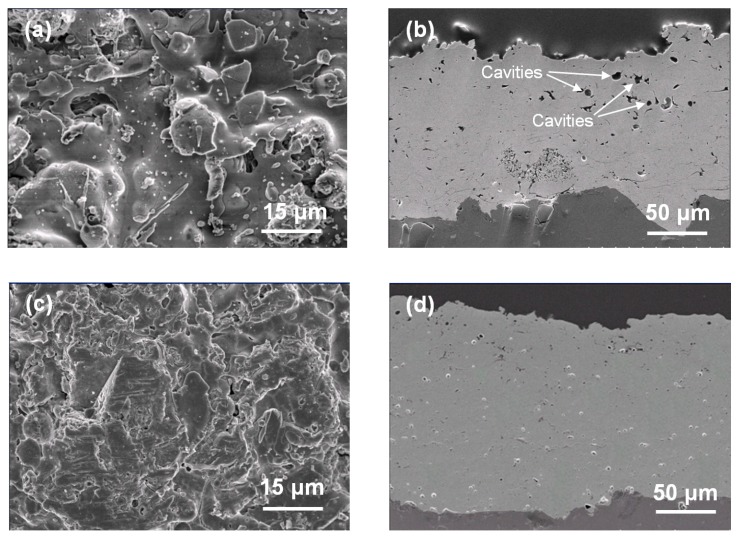
Surface and cross-sectional SEM images of (**a**,**b**) Y_2_O_3_ and (**c**,**d**) YF_3_ coating at plasma spray power of 15 kW.

**Figure 3:**
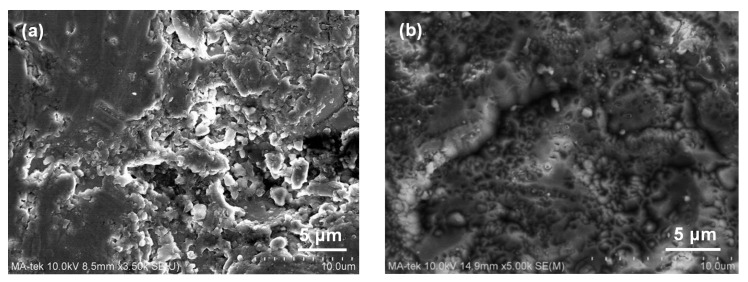
SEM images after fluorocarbon plasma treatment: (**a**) Y_2_O_3_ and (**b**) YF_3_ coatings.

Figure 2 shows the surface and cross-sectional SEM images of Y_2_O_3_ and YF_3_ coatings under 15 kW plasma spraying powers. Figure 2a shows the Y_2_O_3_ coating with poor surface roughness with laminar morphology. A horizontal crack and large cavities (within the size range of 5–10 μm) are also observed (Figure 2b). Dense YF_3_ coating layers with less porosity are shown in Figure 2c,d). Due to the small difference in thermal expansion between the YF_3_ coating (28.5 × 10^−6^/K) and the Al substrate (23 × 10^−6^/K), no cracks were observed in any YF_3_ coating samples. The erosion-resistance characteristics of Y_2_O_3_ and YF_3_ coatings were measured after exposure to the CF_4_/O_2_ plasma, as discussed below. Figure 3a,b show the SEM image surface microstructure of the Y_2_O_3_ and YF_3_ coatings after etching in CF_4_/O_2_ plasma for 60 min under a bias power of 500 W. There is a large difference in etched surface for both coating samples under the same etching condition. In Figure 3a, the surface of Y_2_O_3_ coating is obviously cracked after the etching process. These Y_2_O_3_ creaked pieces might be a particle contamination source during the wafer fabrication process. In Figure 3b, the YF_3_ coating is revealed to have a relatively dense and smooth surface, indicating that the erosion of Y_2_O_3_ coating was more severe than YF_3_ coating after exposure to CF_4_/O_2_ plasma. It is also consistent with our previous study regarding the film porosity [16], since the erosion resistance to CF_4_/O_2_ plasma (chemical reaction) was enhanced by reducing film porosity [17]. From the SEM observation results, the YF_3_ coating revealed that a clean and complete surface can be obtained by preventing the fluoride particles from attaching to the etching chamber sidewall after exposure to the fluorocarbon plasma during etching. Hence, the YF_3_ coating is more favorable for application in plasma processing equipment. Similar results were consistent with the previously reported data by Kim et al. [18].

**Figure 4:**
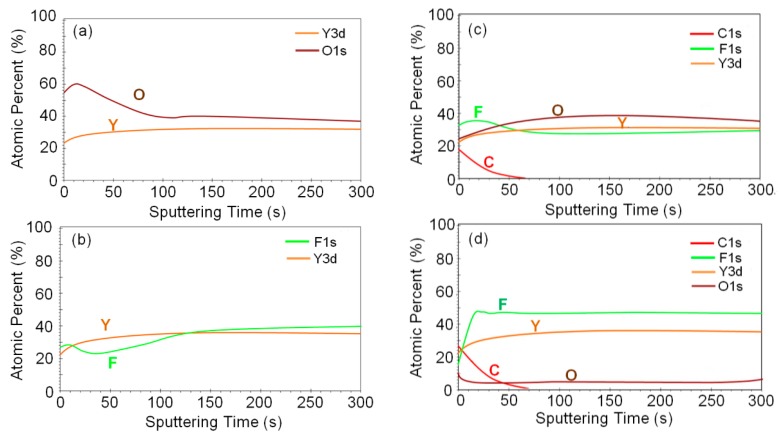
Variations of chemical compositions measured using X-ray photoelectron spectroscopy (XPS) with the sputtering time of (**a**,**c**) the Y_2_O_3_ and (**b**,**d**) the YF_3_ coatings before and after exposure to the fluorocarbon plasma.

Figure 4 shows the compositional variation with the sputtering time from the Y_2_O_3_ and YF_3_ coating surfaces after exposure to the CF_4_/O_2_ plasma by using XPS. The Y_2_O_3_ and YF_3_ coating surfaces before exposure to the CF_4_/O_2_ plasma are shown in Figure 4a,b. The carbon content found on the surface decreased abruptly with the sputtering time, indicating that the carbon polymer layer is very thin (Figure 4c,d). This thin carbon polymer layer on the etched surface was previously reported in Si-based oxide materials etched under fluorocarbon plasma [19,20,21]. Moreover, the fluorine was verified on both Y_2_O_3_ and YF_3_ coatings and the higher fluorine content was detected on the YF_3_ surface (Figure 4c,d). It was found that the percentage of F atoms reached the maximum value of 35% and 48% on the Y_2_O_3_ and YF_3_ coatings after etching, causing a thicker fluorination layer to appear in the YF_3_ specimen. This is because YF_3_ is a fluorine-rich compound material; hence, the chemical composition of F atoms in the YF_3_ coating was unchanged accompanied with the depth from the surface. The stronger YF_3_ coating fluorination can also be confirmed by the XPS spectral analysis on etched sample surfaces.

**Figure 5:**
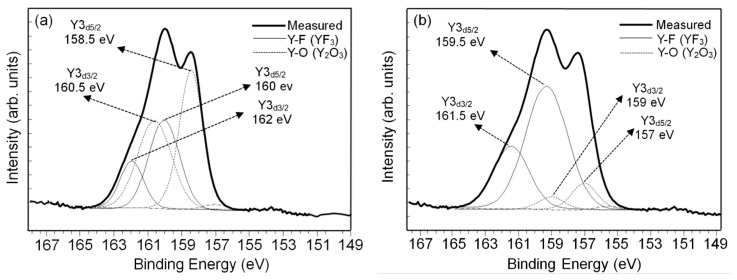
Variations of chemical compositions measured using XPS with the sputtering time of (**a**) the Y_2_O_3_ and (**b**) the YF_3_ coatings after exposure to the fluorocarbon plasma.

Figure 5 represents the XPS spectra for the yttrium atoms from the (a) Y_2_O_3_ and (b) YF_3_ coating samples after CF_4_/O_2_ plasma etching. In the curve-fitted XPS spectra of the Y_2_O_3_ coating, two peaks mean two sources of bonding for cations from Y3d split into two Y3d_5/2_ states (high peaks) and Y3d_3/2_ (low peaks) [22]. In Figure 5a, the etched surface of the Y_2_O_3_ coating consisted of Y3d_5/2_ and Y3d_3/2_, with an intensity ratio of 3:2 and peak shift difference in the binding energy of 2 eV, according to the XPS standard [23,24]. This peak shifting to higher binding energy (located at 160 and 162 eV) could be attributed to Y–F bonding in the Y_2_O_3_ sample. The higher electronegativity of fluorine (4.0) compared to that of oxygen (3.5) causes higher electron binding energy from the cation [25]. This high fluorine concertation demonstrated that the Y_2_O transformed into a YO*_x_*F*_y_* (*x* + *y* = 1.5) and/or YF*_x_* (*x* < 3) surface by the exposed fluorine-based plasma [26]. Meanwhile, the two binding energy peaks located at 158.5 and 160.5 eV correspond to a Y–O bond, resulting in a low binding energy. Figure 5b shows the YF_3_ coating XPS spectra on the etched surface deconvoluted into four peaks, corresponding to Y–F bonding (located at 159.5 and 161.5 eV) and Y–O bonding (located at 157 and 159 eV) [27,28]. Furthermore, the intensity ratio of Y–F to Y–O peaks on the YF_3_ coating reached 2.9, which was much higher than that of the Y–F to Y–O peak on the Y_2_O_3_ coating (0.73), indicating stronger fluorination on the etched YF_3_ coating surface. These results indicate that the YF_3_ coating exhibited superior inherent chemical stability after fluorocarbon plasma treatment [29]. The reactions for CF_4_/O_2_ plasma chemical etching of Y_2_O_3_ and YF_3_ coatings can be expressed as follows:

Y_2_O_3_ + 3CF_2_* → 2YF_3_ + 3CO;

YF_3_ + 2CF_2_* → YF + 2CF_3_.

when the CF_4_/O_2_ plasma chemical reaction dominates the etching process, and the chemical stability YF_3_ might be effective in the suppression of particle generation during the etching process, as mentioned in the SEM results.

**Figure 6:**
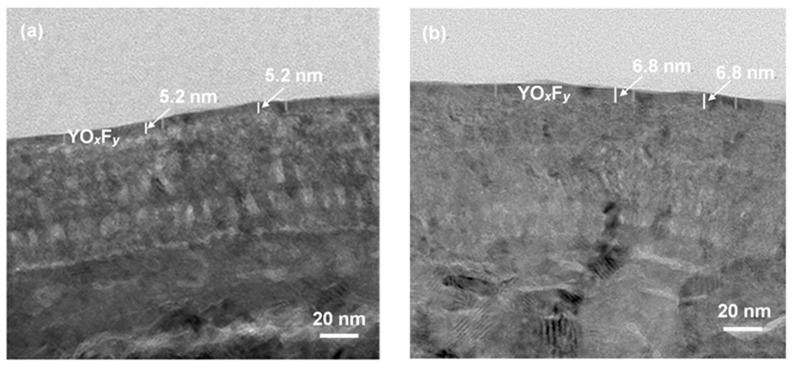
TEM microstructures of (**a**) Y_2_O_3_ and (**b**) YF_3_ coatings after exposure to the fluorocarbon plasma.

The microstructures of both coated samples irradiated by high-density CF_4_/O_2_ plasma were revealed by TEM. Figure 6a,b shows cross-sectional TEM micrographs of the plasma-etched Y_2_O_3_ and YF_3_ coatings, respectively. A yttrium oxyfluoride (YO*_x_*F*_y_*) film 5.2 nm in thickness was observed on the Y_2_O_3_ surface, while the YF_3_ sample showed a thicker YO*_x_*F*_y_* of 6.8 nm. The slightly lesser fluorination of Y_2_O_3_ than YF_3_ is explained by comparing the bonding energies of Y–O (685 KJ/mol) and Y–F (605 KJ/mol). Because the bonding energy of Y–O is higher than Y–F, it results in less-efficient reactions between the Y–O bonding and the fluorocarbon film. The formation of an oxyfluoride layer on the surface of Y_2_O_3_ and YF_3_ coatings might act as a protecting layer to prevent the coating’s surface from further erosion by CF_4_/O_2_ plasma.

**Figure 7:**
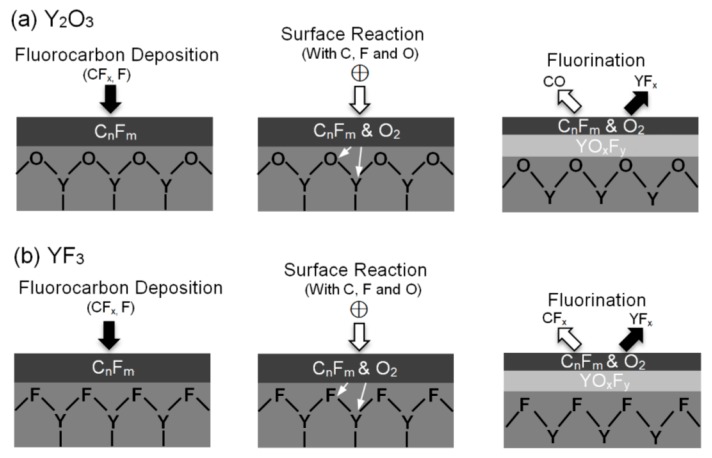
Schematic illustration of yttrium oxyfluoride film deposition behavior on (**a**) Y_2_O_3_ and (**b**) YF_3_ coatings.

Figure 7 illustrates the schematic formation mechanism of YO*_x_*F*_y_* on Y_2_O_3_ and YF_3_ surfaces. The procedure of YO*_x_*F*_y_* formation on the Y_2_O_3_ and YF_3_ surfaces is as follows: the first step is deposition of the fluorocarbon film by the adsorption of CF*_x_* radicals on these two coatings’ sample surfaces. The second step is carbon reactions with oxygen (Y–O) and fluorine (Y–F) to form volatile CO and CF*_x_*, resulting in the decomposition of the Y–O and Y–F bondings. Subsequently, the YO*_x_*F*_y_* film is formed in the Y_2_O_3_ and YF_3_ coatings surface (third step), whereas a part of YF*_x_* may desorb from the coating surface. Similar reactions and formation of YO*_x_*F*_y_* were also investigated previously [30,31]. It is believed that the YO*_x_*F*_y_* layer is effective in reducing practical generation and thus contamination of the integrated circuit [32]. 

**Figure 8:**
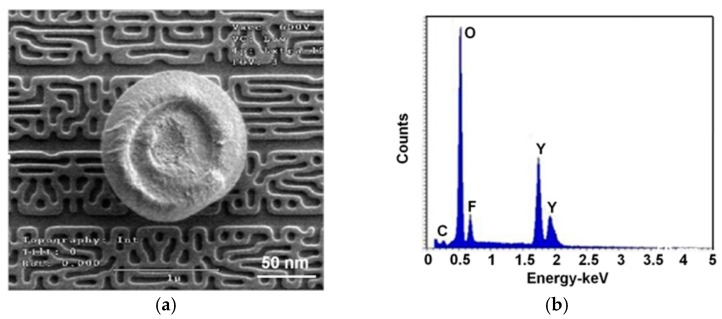
(**a**) SEM image of a circular particle and (**b**) energy dispersive X-ray diffraction (EDX) analysis results of circular particle.

One of the prime concerns of production yields is the particles generated from the plasma processing equipment, resulting in open- or short-circuits. We have investigated the possible particles generated during the etching process using an in-situ particle monitoring system which can detect the particle trajectories. Figure 8a shows an SEM image of a typical particle observed on the wafer surface. The circular particle is composed of etchant gases and a Y_2_O_3_-coated chamber wall after exposed to CF_4_/O_2_ plasma—it can be called a partial etch defect. An EDX analysis was carried out to clarify the particle compositions. As shown in Figure 8b, it was found that the flaking particles were composed mainly of yttrium, oxide, and fluoride elements, indicating the particle source from the Y_2_O_3_ coating. Therefore, the YF_3_ coating can behave as a new plasma-facing material that produces fewer contamination particles.

## 4. Conclusions

During the plasma etching process, particles generated from the Y_2_O_3_ and YF_3_ protective coatings of the ICP chamber wall were investigated in this study. The particle generation mechanism could be due to the fact that the bonding strength of Y–O was weaker than that of Y–F when the chamber-wall surface suffered irradiation from high-density CF_4_/O_2_ plasmas. From the SEM examination results, YF_3_ was also confirmed to be more robust than Y_2_O_3_ against CF_4_/O_2_ plasma irradiation. The YF_3_ coatings for the ICP etching chamber components and materials can play an important role in decreasing the extreme number of particles in the fluorine-based plasma environment.

## Figures and Tables

**Table 1:** Spraying parameters for coatings made of Y_2_O_3_ and YF_3_.

Condition	Y_2_O_3_	YF_3_
Primary gas flow rate (L/min)	45	45
Secondary current (L/min)	6	6
Gun moving rate (cm/s)	10	10
System voltage (V)	50	50
Gun power (kW)	15	15
Stand-off (cm)	10	10

**Table 2:** Plasma etching parameters for Y_2_O_3_ and YF_3_ coatings.

Condition	Y_2_O_3_	YF_3_
RF source power (W)	1300	1300
RF bias power (W)	500	500
Chamber pressure (Pa)	1.06	1.06
CF_4_:O_2_ (sccm)	30:5	30:5
Etching time (min)	60	60
